# Identification of *NF-κB* and *PLCL2* as new susceptibility genes and highlights on a potential role of *IRF8* through interferon signature modulation in systemic sclerosis

**DOI:** 10.1186/s13075-015-0572-y

**Published:** 2015-03-21

**Authors:** Maria Arismendi, Matthieu Giraud, Nadira Ruzehaji, Philippe Dieudé, Eugenie Koumakis, Barbara Ruiz, Paolo Airo, Daniele Cusi, Marco Matucci-Cerinic, Erika Salvi, Giovanna Cuomo, Eric Hachulla, Elisabeth Diot, Paola Caramaschi, Valeria Riccieri, Jérôme Avouac, Cristiane Kayser, Yannick Allanore

**Affiliations:** Paris Descartes University, INSERM U1016, Institut Cochin, Sorbonne Paris Cité, Paris, France; CAPES Foundation, Ministry of Education of Brazil, Brasília, DF 70040-020 Brazil; Paris Diderot University, Rheumatology Department, Hôpital Bichat Claude Bernard, APHP, Paris, France; Paris Diderot University, INSERM U699, Hôpital Bichat Claude Bernard, Paris, France; Rheumatology and Clinical Immunology, Spedali Civili, Brescia, Italy; University of Milano, Department of Medicine, Surgery and Dentistry San Paolo & Genomics and Bioinformatics Platform, Fondazione Filarete, Milan, Italy; Department of Biomedicine & Division of Rheumatology AOUC, Department of Rheumatology AVC, Department of Medicine & Denothe Centre, University of Florence, Florence, Italy; Department of Clinical and Experimental Medicine, Rheumatology Unit, Second University of Naples, Naples, Italy; Université Lille II, Médecine Interne, Lille, France; INSERM U618, IFR 135, CHU Bretonneau, Tours, France; Rheumatology Unit, Azienda Ospedaliera Universitaria Integrata di Verona, Verona, Italy; Division of Rheumatology, Department of Medical Clinic and Therapy, University “Sapienza” of Rome, Rome, Italy; Paris Descartes University, Rheumatology A Department, Cochin Hospital, APHP, Paris, France; Department of Rheumatology, Federal University of São Paulo, São Paulo, Brazil

## Abstract

**Introduction:**

Systemic sclerosis (SSc) and primary biliary cirrhosis (PBC) are rare polygenic autoimmune diseases (AIDs) characterized by fibroblast dysfunction. Furthermore, both diseases share some genetic bases with other AIDs, as evidenced by autoimmune gene pleiotropism. The present study was undertaken to investigate whether single-nucleotide polymorphisms (SNPs) identified by a large genome-wide association study (GWAS) in PBC might contribute to SSc susceptibility.

**Methods:**

Sixteen PBC susceptibility SNPs were genotyped in a total of 1,616 patients with SSc and 3,621 healthy controls from two European populations (France and Italy).

**Results:**

We observed an association between *PLCL2* rs1372072 (odds ratio (OR) = 1.22, 95% confidence interval (CI) 1.12 to 1.33, *P*_adj_ = 7.22 × 10^−5^), nuclear factor-kappa-B (*NF-κB*) rs7665090 (OR = 1.15, 95% CI 1.06 to 1.25, *P*_adj_ = 0.01), and *IRF8* rs11117432 (OR = 0.75, 95% CI 0.67 to 0.86, *P*_adj_ = 2.49 × 10^−4^) with SSc susceptibility. Furthermore, phenotype stratification showed an association between rs1372072 and rs11117432 with the limited cutaneous subgroup (lcSSc) (*P*_adj_ = 4.45 × 10^−4^ and *P*_adj_ = 0.001), whereas rs7665090 was associated with the diffuse cutaneous subtype (dcSSc) (*P*_adj_ = 0.003). Genotype-mRNA expression correlation analysis revealed that the *IRF8* protective allele was associated with increased interferon-gamma (IFN-γ) expression (*P* = 0.03) in patients with SSc but decreased type I IFN (IFIT1) expression in patients and controls (*P* = 0.02). In addition, we found an epistatic interaction between *NF-κB* and *IRF8* (OR = 0.56, 95% CI 0.00 to 0.74, *P* = 4 × 10^−4^) which in turn revealed that the *IRF8* protective effect is dependent on the presence of the *NF-κB* susceptibility allele.

**Conclusions:**

An analysis of pleiotropic genes identified two new susceptibility genes for SSc (*NF-κB* and *PLCL2*) and confirmed the *IRF8* locus. Furthermore, the *IRF8* variant influenced the IFN signature, and we found an interaction between *IRF8* and *NF-κB* gene variants that might play a role in SSc susceptibility.

**Electronic supplementary material:**

The online version of this article (doi:10.1186/s13075-015-0572-y) contains supplementary material, which is available to authorized users.

## Introduction

Systemic sclerosis (SSc) is an autoimmune disease (AID) characterized by vascular damage, autoantibody production, and fibrotic events. SSc is an orphan disease that is considered the most severe connective tissue disorder, representing an important medical challenge because of its debilitating progressive nature [1]. The precise aetiology of the disease remains unclear. However, as in other AIDs, interactions between environment and genetic factors are thought to play a key role in disease susceptibility [2]. Indeed, several reports of well-powered candidate gene studies, together with genome-wide association studies (GWASs), have established numerous SSc susceptibility genes, including *STAT4*, *IRF5*, *TNFSF4*, *CD226*, *CD247*, and *BANK1* [3,4]. The vast majority of these susceptibility loci belong to pathways involved in immune responses or inflammation and were identified in other AIDs [3]. Previous work from our team demonstrated that polyautoimmunity affects up to a quarter of patients with SSc, highlighting the concept of shared autoimmunity in SSc [5]. It is well established that AIDs encompass a broad range of phenotypic manifestations and severity, indicating that the effects of these loci are not of equal magnitude and may not be associated in the same direction (risk or protection) among different diseases [3]. Fibroblast dysfunction, the SSc hallmark, can also be observed in primary biliary cirrhosis (PBC). PBC is a disease marked by progressive destruction of the liver and can co-occur with SSc, suggesting that these two AIDs may share common pathways and may be a result of common-variant loci of weak effect [6]. A recent GWAS carried out in patients with PBC identified 12 new loci involved in disease susceptibility and highlighted the importance of type I interferon (IFN), nuclear factor-kappa-B (NF-*κ*B), and Toll-like receptor (TLR) signaling [7] in its pathogenesis. Given the evidence of shared autoimmune genes between SSc and other AIDs, we investigated the association of 16 single-nucleotide polymorphisms (SNPs), recently identified as susceptibility factors for PBC, with SSc and its subphenotypes.

## Methods

### Study population

We performed a case-control association study with a European Caucasian cohort consisting of 1,616 patients with SSc (1,022 French and 594 Italians) and 3,621 controls (2,384 French and 1,237 Italians). Only individuals with European ancestry (defined as all four grandparents being of European Caucasian ancestry) were included in the study. This cohort has already been used in several previous genetic studies, and high homogeneity between French and Italian samples has been demonstrated [4,8]. Control subjects were age- and sex-matched to patients with SSc. The characteristics of the patients with SSc are shown in Table 1. All patients with SSc were classified by LeRoy’s cutaneous subtype and were phenotypically screened for anti-nuclear antibodies and pulmonary fibrosis (PF). The latter was defined as any of the following changes on computed tomography scan, such as in our previous studies: reticulations, honeycombing, and traction. In line with all of our previous projects, patients with SSc were also evaluated for the presence of other potential AIDs. To avoid any bias due to a possible excess of the risk alleles attributable to these patients, 23 SSc patients showing PBC co-occurrence were excluded from the main study. This study was approved by the Comité Consultatif de Protection des Personnes dans la Recherche Biomédicale (CCPPRB) Paris Cochin (dossier 2068). All participants enrolled in this study provided written informed consent.

**Table 1:** **Characteristics of systemic sclerosis patients included in the study**

	**French cohort (n = 1,022)**	**Italian cohort (n = 594)**	**Combined cohort (n = 1,616)**
Age in years, mean ± SD	57.4 ± 13.9	48.8 ± 13.4	53.1 ± 13.6
Male gender, number	152	55	207
Disease duration in years, mean ± SD	13.1 ± 7.1	12.4 ± 8.7	12.75 ± 7.9
lcSSc, percentage	60	74.7	64.4
dcSSc, percentage	29.5	25.2	28
Lung fibrosis seen on CT, percentage	36.1	34.6	35.3
FVC <75%, percentage	14	16.1	14.6
Digital ulcers (ever occurred), percentage	33	44	36.6
ACA+, percentage	32.3	44.6	36.6
Anti-topo I+, percentage	23	32.6	26.3
Associated AID, percentage	15	14.6	15

ACA, anti-centromere antibody; anti-topo I, anti-topoisomerase antibody; associated AID, at least one associated autoimmune disease; CT, computed tomography; dcSSc, diffuse cutaneous systemic sclerosis; FVC, forced vital capacity; lcSSc, limited cutaneous systemic sclerosis; SD, standard deviation.

### Single-nucleotide polymorphism selection

The SNP selection was based on a previous PBC GWAS, which identified these SNPs as susceptibility genes to PBC [7]. Among these candidates, two SNPs, *STAT4* rs10931468 and *IRF5* rs12531711, located in loci reproducibly strongly associated with SSc in several studies [3,4], were excluded from the study. The list of the selected SNPs, as well as information about their localization and putative function, is shown in Additional file 1: Table S1.

### Genotyping

DNA samples from SSc patients and controls were genotyped for the 16 tag SNPs: rs10752747, rs12134279, rs1372072, rs2293370, rs485499, rs7665090, rs860413, rs6974491, rs6421571, rs1800693, rs911263, rs8017161, rs11117432, rs7208487, rs3745516, and rs968451. Genotyping was performed by using a competitive allele-specific polymerase chain reaction system (KASpar genotyping; KBioscience, Hoddesdon, UK).

### Gene expression in peripheral blood mononuclear cells

To assess a possible genotype-phenotype correlation, we selected the three associated genes—*PLCL2*, *NFKB1*, and *IRF8*—to perform gene expression assays of their mRNA. Moreover, because of compelling data on *IRF8*-associated pathways, we also investigated IFN-γ and type I IFN (IFIT1) mRNA gene expression. We randomly selected peripheral blood nuclear cell (PBMC) samples from 39 patients (who did not receive immunosuppressive drugs) and 24 healthy controls. The total RNA was obtained by using an RNA extraction kit (RNeasy Mini Kit; Qiagen, Hilden, Germany) followed by a c-DNA reverse transcription step (SuperScript II Reverse Transcriptase; Invitrogen, Waltham, MA, USA). Gene expression was performed by using quantitative Real-Time Polymerase Reaction (Universal Master Mix II; Applied Biosystems, Waltham, MA, USA). All primers were obtained by a predesigned gene expression assay (Applied Biosystems).

### Statistical analysis

Statistical analyses were performed by using PLINK (Harvard and MIT version 1.07, available at [9]). Power calculation was assessed by using a two independent binomial module combined with a one-sided Fisher exact test (StatXact 8 software; Cytel, Cambridge, MA, USA). Tests for conformity with Hardy-Weinberg equilibrium were performed by using a standard chi-square test (1 degree of freedom). Homogeneity between the two cohorts was confirmed by using the Breslow-Day method and therefore the combined data were subsequently analyzed by calculating the pooled odds ratios (ORs) by using a Cochran-Mantel-Haenszel test under fixed effects. Individual association analyses of all SNPs were performed by comparing cases and controls with a two-sided Fisher exact test on allelic distribution. All ORs are provided with their 95% confidence intervals (CIs).

To test SNP × SNP epistasis, the allele frequencies were compared in patients and control subjects. The PLCL2*T alleles, NF-kB*G alleles, and IRF8*A alleles were considered binary random variables, and the dependency of disease on pairwise combinations of these covariates was tested with a logistic regression model. The fitted model included each one of two tested factors as first-order components and an interaction term reflecting the extent of the allelic by allelic epistasis. We applied a logistic regression assuming a double-dominant model. Exact calculations of ORs and *P* values were made with Logxact 8 software (Cytel).

We applied the Bonferroni correction although it is very conservative; 16 tag SNPs were considered to test the association between each SNP and SSc (*P* values were therefore multiplied by 16 to get the adjusted ones). For disease phenotypes correction, we took into account seven subtypes (*P* values were multiplied by 7 to get the adjusted ones). An unpaired two-sided *t* test was used for mRNA expression levels analysis. An adjusted *P* value of less than 0.05 was considered statistically significant.

## Results

### Single-marker analysis

Sixteen SNPs were genotyped in two European populations (French and Italian). Among the SNPs of interest, minor allele frequencies (MAFs) range from 11% to 44%; therefore, we provide a power calculation for these two MAFs. For an 11% MAF, our power to detect an association is more than 99% for an OR of 1.5 and is 81% to detect an OR of 1.3. For a 44% MAF, our power is more than 99% to detect an OR of 1.3 or higher. Genotype frequencies were in Hardy-Weinberg equilibrium in the control population for all of the SNPs investigated.

We first evaluated the French sample and observed six loci for which a nominal *P* value was reached: *PLCL2* rs1372072 OR = 1.22, 95% CI 1.10 to 1.36, *P* = 1.97 × 10^−4^; *TIMMDC1* rs2293370 OR = 0.84, 95% CI 0.73 to 0.96, *P* = 1.46 × 10^−2^; *IL12A* rs485499 OR = 0.88, 95% CI 0.79 to 0.99, *P* = 3.37 × 10^−2^; *NF-kB* rs7665090 OR = 1.12, 95% CI 1.01 to 1.25, *P* = 2.47 × 10^−2^; *IL7R* rs860413 OR = 0.89, 95% CI 0.79 to 1.00, *P* = 5.77 × 10^−2^; and *IRF8* rs11117432 OR = 0.68, 95% CI 0.68 to 0.93, *P* = 6.52 × 10^−3^. We then analysed all the SNPs in the Italian sample and observed that five SNPs, including three previously identified in the French sample, were associated with SSc: *PLCL2* rs1372072 OR = 1.22, 95% CI 1.05 to 1.41, *P* = 7.76 × 10^−3^; *NF-kB* rs7665090 OR = 1.20, 95% CI 1.04 to 1.38, *P* = 9.00 × 10^−3^; *ELMO1* rs6974491 OR = 1.27, 95% CI 1.04 to 1.55, *P* = 1.93 × 10^−2^; *RAD51B* rs911263 OR = 0.80, 95% CI 0.69 to 0.93, *P* = 4.18 × 10^−3^; and *IRF8* rs11117432 OR = 0.70, 95% CI 0.57 to 0.85, *P* = 5.48 × 19^−4^. It is of note that, when Bonferroni correction was applied in each population, only *PLCL2* rs1372072 *P* = 3 × 10^−3^ and *IRF8* rs11117432 OR = 0.68, 95% CI 0.68 to 0.93, *P* = 8.76 × 10^−3^ remained significant in French and Italian samples, respectively (Table 2).

**Table 2:** **Analysis of** ***PLCL2*** **rs1372072,** ***NF-κB*** **rs7665090, and** ***IRF8*** **rs11117432 gene variants in French and Italian populations**

**SNP, phenotype (n)**	**MAF**	**Genotype distribution**			***P*** **value**	***P***_**adj**_^**a**^	**OR (95% CI)**
French Caucasian							
*PLCL2* rs1372072	T	TT (%)	TC (%)	CC (%)			
SSc (1,021)	0.39	15.40	47.41	37.17	1.97 × 10^−4^	3.15 × 10^−3^	1.22 (1.10-1.36)
dcSSc (298)	0.39	15.64	46.93	37.41	0.02	0.39	1.22 (1.03-1.46)
SSc. Topo I+ (233)	0.39	15.51	48.27	36.20	0.02	0.39	1.25 (1.03-1.52)
lcSSc (591)	0.38	15.29	47.07	37.62	4.45 × 10^−3^	0.07	1.21 (1.06-1.38)
SSc. ACA+ (349)	0.39	16.90	45.77	37.31	5.37 × 10^−3^	0.08	1.26 (1.07-1.49)
Pulmonary fibrosis (364)	0.40	16.62	48.47	34.90	6.97 × 10^−4^	0.01	1.32 (1.12-1.55)
Controls (2,384)	0.34	12.31	44.01	43.67	NA	NA	NA
*NF-κB* rs7665090	G	GG (%)	AG (%)	AA (%)			
SSc (1,021)	0.52	27.21	49.59	23.18	0.02	0.39	1.12 (1.01-1.25)
dcSSc (298)	0.54	32.29	44.79	22.91	0.01	0.16	1.25 (1.05-1.49)
SSc. Topo I+ (233)	0.54	31.14	47.36	21.49	0.01	0.29	1.26 (1.04-1.53)
lcSSc (591)	0.50	24.95	51.81	23.22	0.26	NS	1.07 (0.94-1.22)
SSc. ACA+ (349)	0.50	23.97	52.04	23.97	0.65	NS	1.04 (0.88-1.22)
Pulmonary fibrosis (364)	0.52	28.53	48.87	22.59	0.05	0.84	1.17 (1.00-1.37)
Controls (2,384)	0.49	23.35	51.31	25.33	NA	NA	NA
*IRF8* rs11117432	A	AA (%)	AG (%)	GG (%)			
SSc (1,021)	0.16	2.53	27.28	70.18	6.52 × 10^−3^	0.10	0.79 (0.68-0.93)
dcSSc (298)	0.15	2.38	25.25	72.35	0.01	0.22	0.73 (0.56-0.94)
SSc. Topo I+ (233)	0.16	3.52	26.87	69.60	0.23	NS	0.84 (0.64-1.10)
lcSSc (591)	0.16	2.28	29.22	68.48	0.08	NS	0.84 (0.69-1.01)
SSc. ACA+ (349)	0.15	3.28	25.37	71.34	0.04	0.74	0.78 (0.62-0.99)
Pulmonary fibrosis (364)	0.16	2.26	27.76	69.97	0.05	0.82	0.79 (0.63-1.00)
Controls (2,384)	0.19	3.77	31.33	64.88	NA	NA	NA
Italian Caucasian							
*PLCL2* rs1372072	T	TT (%)	TC (%)	CC (%)			
SSc (607)	0.36	13.36	47.03	39.59	7.76 × 10^−3^	0.12	1.22 (1.05-1.41)
dcSSc (152)	0.34	11.72	46.20	42.06	0.42	NS	1.11 (0.86-1.44)
SSc. Topo I+ (233)	0.35	11.57	47.36	41.05	0.29	NS	1.13 (0.90-1.42)
lcSSc (455)	0.37	13.90	47.30	38.78	5.55 × 10^−3^	0.08	1.25 (1.06-1.47)
SSc. ACA+ (349)	0.38	14.66	48.49	36.84	4.49 × 10^−3^	0.07	1.32 (1.09-1.61)
Pulmonary fibrosis (210)	0.39	13.72	50.98	35.29	7.62 × 10^−3^	0.12	1.34 (1.08-1.67)
Controls (1,237)	0.32	10.68	43.41	45.89	NA	NA	NA
*NF-κB* rs7665090	G	GG (%)	AG (%)	AA (%)			
SSc (607)	0.52	29.66	47.45	24.23	9.00 × 10^−3^	0.14	1.20 (1.08-1.33)
dcSSc (152)	0.55	33.33	44.02	22.66	0.01	0.31	1.34 (1.03-1.76)
SSc. Topo I+ (233)	0.50	24.61	51.79	23.58	0.38	NS	1.10 (0.81-1.37)
lcSSc (455)	0.51	27.90	47.76	24.33	0.05	0.89	1.16 (0.96-1.34)
SSc. ACA+ (349)	0.51	27.50	47.58	24.90	0.18	NS	1.14 (0.95-1.34)
Pulmonary fibrosis (210)	0.56	34.61	42.78	22.59	2.29 × 10^−3^	0.04	1.37 (1.17-1.68)
Controls (1,237)	0.48	22.60	50.85	26.53	NA	NA	NA
*IRF8* rs11117432	A	AA (%)	AG (%)	GG (%)			
SSc (607)	0.12	1.17	23.23	75.58	5.47 × 10^−4^	8.76 × 10^−3^	0.70 (0.57-0.85)
dcSSc (152)	0.14	2.64	24.50	72.84	0.33	NS	0.83 (0.60-1.17)
SSc. Topo I+ (233)	0.12	2.04	21.42	76.53	0.02	0.44	0.70 (0.51-0.96)
lcSSc (455)	0.12	0.67	22.79	76.52	2.51 × 10^−4^	4 × 10^−3^	0.65 (0.52-0.82)
SSc. ACA+ (349)	0.13	1.14	25.47	73.38	0.06	0.97	0.77 (0.59-1.01)
Pulmonary fibrosis (210)	0.12	1.92	22.11	75.96	0.03	0.51	0.71 (0.52-0.96)
Controls (1,237)	0.17	3.15	28.21	68.63	NA	NA	NA

^a^After Bonferroni correction. ACA+, anti-centromere antibody; CI, confidence interval; dcSSc, diffuse cutaneous systemic sclerosis; lcSSc, limited cutaneous systemic sclerosis; MAF, minor allele frequency; n, number of pooled patients analysed; NA, not applicable; NS, not significant; OR, odds ratio; SNP, single-nucleotide polymorphism; SSc, systemic sclerosis; Topo I+, anti-topoisomerase I antibody.

Therefore, to increase the statistical power, we moved for combined analyses taking into account that homogeneity of the cohorts was confirmed by the Breslow-Day test (no evidence of interpopulation heterogeneity) (Table 3). Our combined analysis revealed a significant association for three of the studied SNPs: *PLCL2* rs1372072 OR = 1.22, 95% CI 1.12 to 1.33, *P*_adj_ = 7.22 × 10^−5^; *NF-kB* rs7665090 OR = 1.15, 95% CI 1.06 to 1.25, *P*_adj_ = 0.01; and *IRF8* rs11117432, OR = 0.75, 95% CI 0.67 to 0.86, *P*_adj_ = 2.49 × 10^−4^ (Table 3). It is noteworthy that combined analyses revealed the same directions of effect for the three SNPs and, of the most importance, that the combined *P* value was greater than the *P* value observed in a single population, altogether, this supports a true association.

**Table 3:** **Analysis of** ***PLCL2*** **rs1372072,** ***NF-κB*** **rs7665090, and** ***IRF8*** **rs11117432 gene variants in the combined Caucasian populations (French and Italian)**

**SNP, phenotype (n)**	**MAF**	**Genotype distribution**			***P*** **value**	***P***_**adj**_^**a**^	**OR (95% CI)**
*PLCL2* rs1372072	T	TT (%)	TC (%)	CC (%)			
SSc (1,597)	0.38	14.65	47.27	38.07	4.01 × 10^−6^	7.22 × 10^−5^	1.22 (1.12-1.33)
dcSSc (439)	0.38	14.35	46.69	38.95	0.01	0.12	1.19 (1.03-1.37)
SSc, Topo I+ (422)	0.38	13.74	47.86	38.38	0.01	0.09	1.20 (1.03-1.39)
lcSSc (1,028)	0.38	14.68	47.17	38.13	6.37 × 10^−5^	4.45 × 10^−4^	1.23 (1.11-1.36)
SSc, ACA+ (609)	0.39	15.92	46.96	37.11	6.21 × 10^−5^	4.35 × 10^−4^	1.29 (1.13-1.46)
Pulmonary fibrosis (565)	0.40	15.57	49.38	35.04	1.29 × 10^−5^	9.07 × 10^−5^	1.33 (1.17-1.51)
Controls (3,570)	0.33	11.76	43.80	44.42	NA	NA	NA
*NF-κB* rs7665090	G	GG (%)	AG (%)	AA (%)			
SSc (1,590)	0.52	27.98	48.55	23.45	7.19 × 10^−4^	0.01	1.15 (1.06-1.25)
dcSSc (438)	0.55	32.64	44.52	22.83	4.93 × 10^−4^	0.003	1.28 (1.11-1.47)
SSc, Topo I+ (423)	0.53	28.13	49.40	22.45	0.01	0.12	1.18 (1.03-1.37)
lcSSc (1,025)	0.51	26.24	50.04	23.70	0.03	0.24	1.11 (1.00-1.22)
SSc, ACA+ (611)	0.51	25.53	50.08	24.38	0.20	NS	1.08 (0.95-1.22)
Pulmonary fibrosis (562)	0.54	30.78	46.61	22.59	7.14 × 10^−4^	0.004	1.24 (1.09-1.41)
Controls (3,585)	0.48	23.09	51.15	25.74	NA	NA	NA
*IRF8* rs11117432	A	AA (%)	AG (%)	GG (%)			
SSc (1,500)	0.14	2.03	25.76	72.22	1.56 × 10^−5^	2.49 × 10^−4^	0.75 (0.67-0.86)
dcSSc (444)	0.15	2.47	25	72.52	0.009	0.06	0.76 (0.62-0.93)
SSc, Topo I+ (423)	0.15	2.83	24.34	72.81	0.01	0.11	0.77 (0.63-0.95)
lcSSc (1,011)	0.14	1.58	26.40	72.00	1.96 × 10^−4^	0.001	0.75 (0.65-0.87)
SSc, ACA+ (598)	0.15	2.34	25.41	72.24	0.005	0.04	0.78 (0.65-0.93)
Pulmonary fibrosis (561)	0.15	2.13	25.66	72.19	0.004	0.02	0.76 (0.63-0.91)
Controls (2,290)	0.18	3.44	29.69	66.85	NA	NA	NA

^a^After Bonferroni correction. ACA+, anti-centromere antibody; CI, confidence interval; dcSSc, diffuse cutaneous systemic sclerosis; lcSSc, limited cutaneous systemic sclerosis; MAF, minor allele frequency; n, number of pooled patients analysed; NA, not applicable; NS, not significant; OR, odds ratio; SNP, single-nucleotide polymorphism; SSc, systemic sclerosis; Topo I+, anti-topoisomerase I antibody.

The next step was to investigate possible associations between *PLCL2*, *IRF8*, and *NF-κB* polymorphisms with clinical features. For that purpose, we stratified the patients according to their main subphenotypes in the combined sample. After Bonferroni correction for multiple testing, we found that the variants *PLCL2* rs1372072 and *IRF8* rs11117432 were associated with the limited cutaneous subgroup (lcSSc) (OR = 1.23, 95% CI 1.11 to 1.36, *P*_adj_ = 4.45 × 10^−4^ and OR = 0.75, 95% CI 0.65 to 0.87, *P*_adj_ = 0.001, respectively) and with the presence of anti-centromere antibodies (ACAs) (OR = 1.29, 95% CI 1.13 to 1.46, *P*_adj_ = 4.35 × 10^−4^ and OR = 0.78, 95% CI 0.65 to 0.93, *P*_adj_ = 0.04) (Table 3).

The *NF-κB* variant rs7665090 was shown to be associated with patients belonging to a diffuse cutaneous phenotype (dcSSc) (OR = 1.28, 95% CI 1.11 to 1.47, *P*_adj_ = 0.003) and also with the PF subset (OR = 1.24, 95% CI 1.09 to 1.41, *P*_adj_ = 0.004) (Table 3). No association was found between the *DENND1B*, *TIMMDC1*, *IL7R*, *ELMO1*, *DDX6*, *TNFRSF1A*, *RAD51B*, *TNFAIP2*, *SPIB*, and *SNORD43* gene variants and SSc (Additional file 2: Table S2).

When the 23 SSc-PBC patients were analysed separately, no significant association was observed when they were compared with the 3,621 controls for any SNPs; furthermore, their addition to the whole sample (23 SSc-PBC + 1,628 SSc patients) did not modify the results (significant association for *PLCL2*, *IRF8*, and *NF-κB*, with unchanged magnitude of the effects). In regard to the group of SSc patients having another AID (n = 224), single-marker analyses revealed significant association with *PLCL2* rs1372072 only (*P* = 0.001; OR = 1.375, 95% CI 1.13 to 1.67).

### Follow-up of the raised hypotheses for single markers

SNPs belonging to the genes highlighted herein have been previously reported as associated or with a trend for association in previous works. For some genes highlighted in our study, some variants not primarily herein studied have been reported as associated with SSc or as having a trend for association with SSc. Indeed, Martin *et al*. [10] reported a suggestive association of *NF-κB* rs1598859, and a robust association has been reported for *IRF8* rs11642873 [11]. Furthermore, a recent work did suggest some association in two loci for which we herein identified a nominal association but not reaching significance after multi-test correction as we observe for DDX6 rs7130875 and IL12A rs77583790 [12].

In our sample, we therefore investigated these markers and compared the association with the one observed with our newly identified SNPs. To that end, we used a subset of our patients for whom we have performed genome-wide typing in the past (sample of 564 patients with SSc and 488 healthy controls) [13]. In regard to NF-κB, we first observed in HapMap that rs1598859 and rs7665090 are not in complete linkage disequilibrium (LD) (D′ = 0.77 and R^2^ = 0.31; SNPs are on the same block according to D′ value but are not in phase according to R^2^) and then found from our GWAS data that the MAFs were 0.355 in patients with SSc and 0.382 in controls (uncorrected *P* value = 0.2). For *IRF8*, an established locus, we first observed that rs11642873 and rs11117432 are not in complete LD (D′ = 0.707 and R^2^ = 0.456). Within our subset with GWAS data, we identified that the rs11642873 MAFs were 0.14 in the patients with SSc and 0.174 in the controls (*P* value = 0.034 without correction; OR = 0.772, 95% CI 0.61 to 0.98). The *P* value is less significant than the one obtained for rs11117432 in the same subset (MAF = 0.156 versus 0.213; *P* = 0.0008), and the OR also shows a weaker effect (OR = 0.68, 95% CI 0.55 to 0.85). We also performed haplotype analyses to estimate the effects of the two SNPs and that relates to conditional analyses; we observed that the haplotype combination including the single rs11642873 risk variant (CG haplotype) was not associated with SSc risk (frequency of 0.108 versus 0.117; *P* = 0.499) but that the haplotype combination including the single rs11117432 risk variant (AA haplotype) was significantly associated with SSc risk (0.124 versus 0.156; *P* = 0.032). We proceeded similarly for *DDX6* rs7130875 and observed that it was not in LD with rs6421571 (D′ = 0.839 and R^2^ = 0.084). DDX6 rs7130875 was not included in our array, but we used the perfect proxy rs4499035 and identified that the MAFs were 0.261 in patients with SSc and 0.252 in the controls (uncorrected *P* value = 0.69). *IL12A* rs77583790 SNP was not included in the array used in our GWAS, and no accurate imputation could be done. Therefore, we have performed a dedicated genotyping by using the TaqMan method in a subset of the patients and controls for whom DNA was still available and who did participate in the present study; we found that rs77583790 minor A allele was present in 2.33% of 1,585 patients with SSc and in 1.36% of 1,842 healthy controls showing a nominal association but not supporting any correction for multiple testing (uncorrected *P* = 0.04). We have thereafter performed some meta-analysis by using the data included in the cited works [10-12] together with ours, thanks to Pr Javier Martin, who provided the genotypic data from his original studies (the sample size was not always the same as in the original study which could include several cohorts from various origins). As shown in Additional file 3: Table S3, neither the *P* value nor the OR was improved for *NF-κB*, *DDX6*, and *IL12A* genes. This suggests that adding our data does not strengthen the previous findings and does not support a significant association for *NF-κB rs1598859* and *DDX6 rs7130875*. In regard to IL12A rs77583790, our results do not provide an independent replication; however, the meta-analysis shows a more significant *P* value when the French population is added, suggesting that this locus is further confirmed by our data. For *IRF8* rs11642873, our data decrease the *P* value, and although the OR is unchanged, the CI is reduced, suggesting that the association is both stronger and of higher magnitude (Additional file 3: Table S3).

### Joint effects of *PLCL2*, *NF-κB*, and *IRF8* risk alleles on systemic sclerosis susceptibility

**Figure 1:**
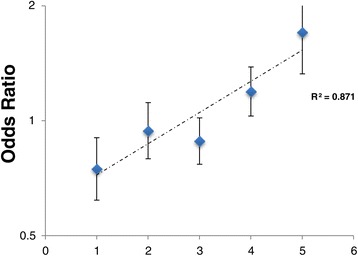
**Joint effects analysis of** ***IRF8*** **,** ***NF-κB*** **, and** ***PLCL2*** **risk alleles on systemic sclerosis (SSc) susceptibility.** Joint effects of *PLCL2* rs1372072, *NF-κB* rs7665090, and *IRF8* rs11117432 risk alleles on SSc susceptibility. Values for the number of risk alleles are taken randomly amongst the genotypes, meaning that a homozygous patient for one single-nucleotide polymorphism counts for two alleles even if only one gene is represented. The results of a linear regression analysis show a multiplicative effect of the alleles on SSc susceptibility. The odds ratios (ORs) with 95% confidence intervals are shown as a function of the number of risk alleles of SSc. The slope of the line corresponds to a 1.20-fold increase in the OR for each additional risk allele. (OR is provided as a log2 scale).

We then investigated the joint effects of *PLCL2* rs1372072*T, *NF-κB* rs7665090*G, and *IRF8* rs1117432*A risk alleles on SSc susceptibility. The ORs for SSc were 0.74 (95% CI 0.62 to 0.90) for carriers of one or two risk alleles, 0.94 (95% CI 0.79 to 1.11) for carriers of three risk alleles, 0.88 (95% CI 0.77 to 1.01) for carriers of four alleles, 1.19 (95% CI 1.03 to 1.38) for carriers of five alleles, and 1.70 (95% CI 1.32 to 2.19) for carriers of six risk alleles. Figure 1 shows the ORs for SSc patients with one or two and three to six risk alleles. Our results demonstrate that the risk of SSc increases proportionally as the number of alleles increases, with an OR of 1.70 when six risk alleles are considered (Figure 1).

### Genetic interaction between associated single-nucleotide polymorphisms

**Figure 2:**
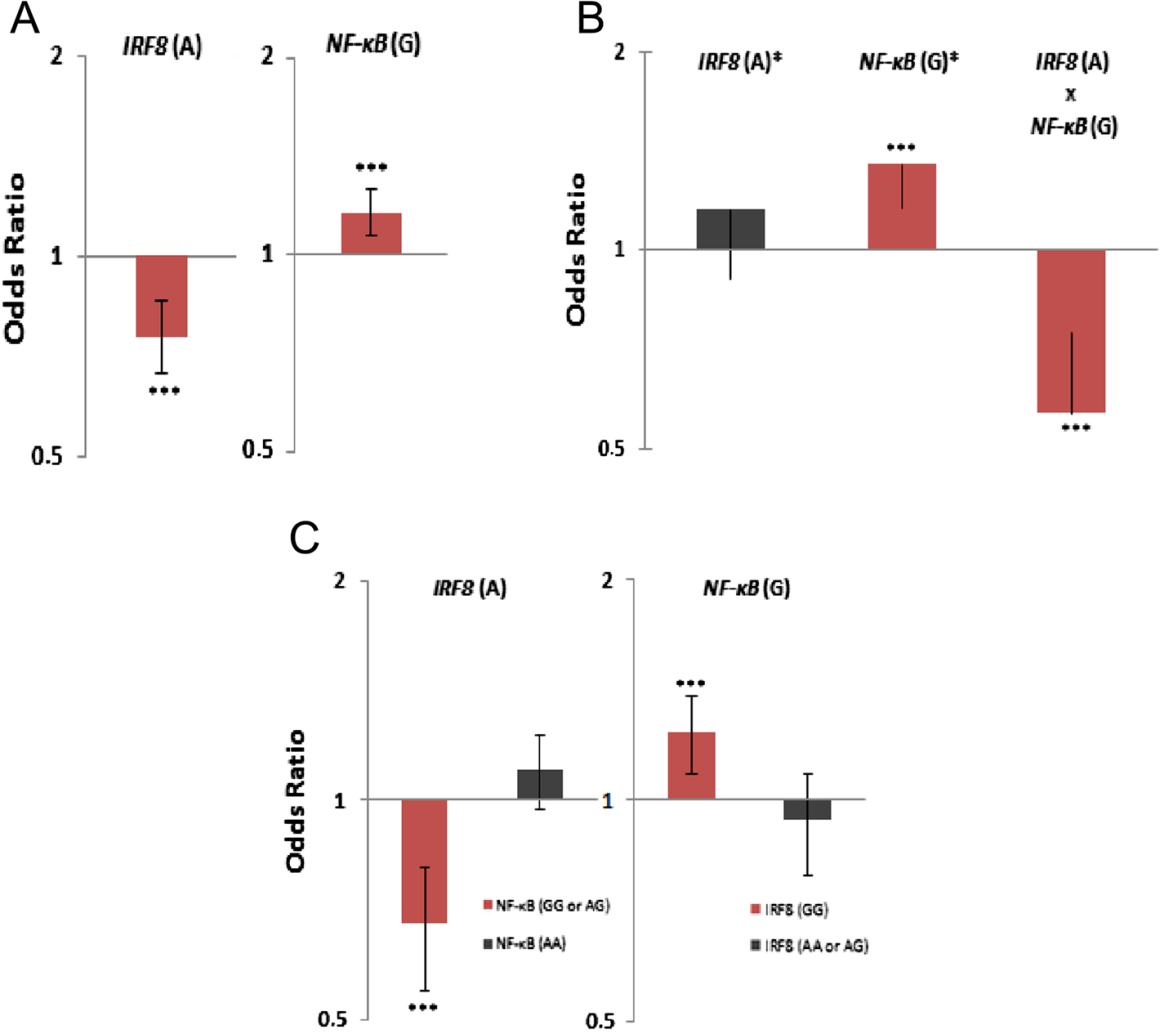
**Analysis of the interaction between** ***IRF8*** **(A) and** ***NF-κB*** **(G) alleles. (A)** Odds ratios (ORs) and 95% confidence intervals attributed to *IRF8* rs11117432 A and *NF-κB* rs7665090 G single-nucleotide polymorphisms (SNPs) after systemic sclerosis (SSc) association analysis. **(B)** Individual effect of *IRF8* A and *NF-κB* G compared with the combined effect of both SNPs after logistic regression analysis, demonstrating that the protective effect of *IRF8* A is observed only when *NF-κB* G is present. The black column represents the group of patients carrying *IRF8* (A) allele who did not carry *NF-kB* (G) allele, indicating a risk effect for *IRF8* (A) (OR = 1.15). The grey column represents patients only harboring *NF-kB* (G) allele (OR = 1.35). The red column represents the group of patients who carried *IRF8* (A) and *NF-kB* (G) at the same time, indicating that the protective effect of *IRF8* (A) is observed only when *NF-κB* (G) is present (OR = 0.56). **(C)** Analysis of the effect of the susceptible alleles in patients stratified by the presence of *NF-KB* G or *IRF8* A. (OR is provided as a log2 scale.) ***P <0.05.

To investigate possible gene-gene interactions between the associated SNPs, we applied a logistic regression (multivariate analysis) on *PLCL2* rs1372072*T, *NF-κB* rs7665090*G, and *IRF8* rs1117432*A alleles. Of the most interest, we found an epistatic interaction between *IRF8* and *NF-κB* (OR = 0.56, 95% CI 0.00 to 0.74, *P* = 4 × 10^−4^), which in turn demonstrated that the protective effect of the *IRF8* A allele is revealed when the *NF-κB* G allele is present (Figure 2A and B). Indeed, when patients were stratified according to the presence of susceptibility alleles A and G, we confirmed that the *IRF8* A protective effect is dependent on the presence of the *NF-κB* G allele (*NF-κB* GG or AG: OR = 0.66, 95% CI 0.57 to 0.77, *P* = 5.91 × 10^−8^ and *NF-κB* AA: OR = 1.10, 95% CI 0.85 to 1.41, *P* = 0.45). Consistent with that, *NF-κB* risk OR was observed only in patients without the protective allele of *IRF8* A (*IRF8* GG: OR = 1.23, 95% CI 1.10 to 1.38, *P* = 1 × 10^−4^ and *IRF8* AA or AG: OR = 0.93, 95% CI 0.79 to 1.11, *P* = 0.46) (Figure 2C).

### mRNA expression

**Figure 3:**
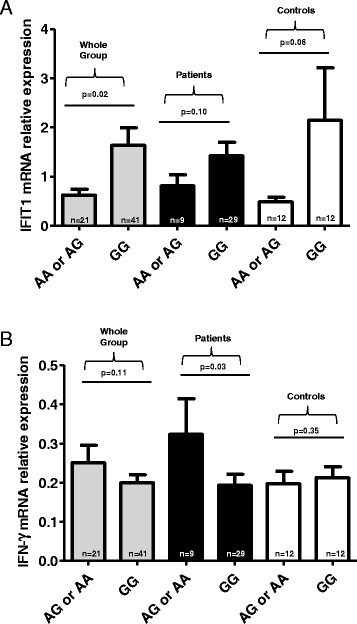
**Type I interferon (IFIT1) and interferon-gamma (IFN-γ) mRNA expression levels in patients with systemic sclerosis (SSc) and healthy controls. (A)** Influence of *IRF8* genotype on IFIT1 mRNA expression in peripheral blood nuclear cells (PBMCs) from patients with SSc and healthy controls (mean ± standard deviation values; uncorrected *P* values). Expression of IFIT1 mRNA was decreased in carriers of the rs11117432 A allele as compared with non-carriers of the A allele. Vertical bars indicate the means. **(B)** Influence of *IRF8* genotype on IFN-γ mRNA expression in PBMCs from patients with SSc and healthy controls. Expression of IFN-γ mRNA was increased in SSc patient carriers of the rs11117432 A allele as compared with non-carriers of the A allele. Vertical bars indicate the means.

We then investigated the potential influence of the three associated gene variants *PLCL2*, *NF-κB*, and *IRF8* on their mRNA expression levels in 38 patients with SSc and 24 controls. After stratification according to the various genotypes, we observed no genotype-phenotype correlation. However, we further analyzed a possible effect of the variant *IRF8* rs11117432 on IFIT1 and IFN-γ mRNA relative expression in the same 38 patients with SSc (nine of them with the *IRF8* rs11117432 AA or GA genotypes and 29 with the GG genotype) and 24 controls (12 with the *IRF8* rs11117432 AA or GA genotypes and 12 with the GG genotype) according to the published data on *IRF8* effects on IFN signature. Using a multiple linear regression test, we found out that the *IRF8* rs11117432*A allele was associated with IFIT1 mRNA expression levels independently of the status of the included individuals (*P* = 0.022, data not shown). When all individuals were considered, our results demonstrated that the *IRF8* rs11117432*A variant was associated with decreased levels of IFIT1 mRNA expression (*P* = 0.02). When patients and controls were considered separately, a trend was observed in the comparison of the *IRF8* rs11117432*A carriers with the non-carriers (*P* = 0.10 for patients, *P* = 0.06) (Figure 3A). We also observed increased levels of IFN-γ mRNA expression in SSc patient carriers of *IRF8* rs11117432*A when compared with the non-carriers (*P* = 0.03) with a trend for difference for the whole group (*P* = 0.11) but no difference in controls (*P* = 0.35) (Figure 3B). There was no effect of the epistatic interaction on IFIT1 or IFN-γ expression (*P* = 0.59 for IFIT1 and *P* = 0.50 for IFN-γ).

## Discussion

The present study identified *NF-κB* and *PLCL2* as new SSc susceptibility genes and confirmed the *IRF8* locus. We also showed potential effects on the IFN signature in regard to the *IRF8* gene variant and an interaction between *IRF8* and *NF-κB* genes.

Our results corroborate previously reported associations in regard to the *IRF8* gene and SSc susceptibility. Here, we demonstrate, for the first time, the specific association between *IRF8* rs11117432 SNP and SSc susceptibility and provide data suggesting a stronger association for rs11117432 as compared with rs11642873, although our meta-analysis results strengthen the previous findings for this marker [11]. Indeed, our haplotype analyses further confirm a stronger association for rs11117432 for a subset of our patients, but a denser genotyping would be required to better narrow a potential causal variant. *IRF8* is a nuclear protein, which upon stimulation by pathogen-associated molecular pattern molecules (PAMPs) moves into the cytoplasm and activates NF-κB and TLR signaling pathways, leading to cytokine production and thereby regulating inflammatory responses [14]. The consequences of unregulated inflammation are associated with the development of pathologic fibrosis such as that seen in SSc [15]. Here, we show that the variant *IRF8* rs11117432 increases IFN-γ but decreases IFIT1 gene expression. Despite reaching significance, our results show subtle differences, and given the complex regulation of protein synthesis and activity, more work is required to confirm the functional impacts of the *IRF8* variant. IFNs are a group of cytokines that play an important role in the regulation of inflammation [16]. Type I IFNs, such as IFIT1, seem to contribute to the upregulation of TLR-induced inflammation and transforming growth factor-beta (TGF-β)-responsive genes expressed by fibroblasts, whereas IFN-γ is a potent suppressor of the pro-inflammatory TGF-β signaling pathway, which in turn reduces the production of collagen I synthesis critical to sustain profibrotic processes in the extracellular matrix [17-19]. Therefore, our results are in agreement with the role played by those specific cytokines and support the protective role of the *IRF8* rs11117432 variant. Thus, *IRF8* polymorphisms might have an effect on pro- and anti-inflammatory modulation, along with regulation of the extracellular matrix, and collagen deposition in fibrotic diseases. A recent study of serologic and cytokine profiles in patients with systemic lupus erythematosus also demonstrated an association between an *IRF8* variant (rs17445836) and decreased levels of IFIT1 [20]. Moreover, according to HapMap data, the SNPs rs11117432 and rs17445836 are located in a regulatory region, underlying potential functional effects of those SNPs. In SSc, *IRF8* SNPs rs11642873 and rs2280381 were already reported as being associated with disease susceptibility. It must be pointed out that rs11117432 and rs2280381 belong to the same block of LD, suggesting a strong association between the *IRF8* region and SSc [11,21]. Of interest, we also identified that the association was preferentially observed in the lcSSc subset and its usual associated anti-nuclear auto-antibody (anti-centromere), suggesting a potential role for the pathogenesis in this subgroup of patients. Epidemiological studies indicate that lcSSc and anti-centromere-positive patients are often associated with other AIDs, including secondary Sjögren thyroiditis and PBC, where IFIT1 is known to play a critical role [5,6].

As a complex polygenic AID, SSc is thought to have innumerous genes play a role in its aetiology and heterogeneity. However, only a handful of genes have been identified as SSc risk variants. Moreover, these variants confer relatively small increments in risk and might explain only a small proportion of the trait heritability, suggesting that other loci remain to be discovered. Here, we describe for the first time the association between *NF-κB* rs7665090 and SSc susceptibility. A previous work suggested that *NF-κB* rs1598859 had a suggestive association with SSc [10]; our results do support such an association after the analyses of a subset of our sample and by the performance of a meta-analysis combining our results together with the previously reported ones [10]. NF-κB is present in the cytoplasm of virtually all cell types in an active form associated with the inhibitory κB (IκB) and has a key role in primarily immune responses related to antigen recognition, being commonly associated with the development of autoimmune and chronic inflammatory diseases [22]. Of interest, the dysregulation of NF-κB activity has already been implicated in the pathogenesis of systemic lupus erythematosus [23]. In SSc, the functional implications of *NF-κB* polymorphisms are still unclear. The analysis of *NF-κB* rs7665090 variant demonstrated no effect on the levels of NF-κB mRNA expression; this is a false-negative result, which may have been due to the small sample size of PBMCs obtained from individuals with SSc. Further studies with a larger sample size might better elucidate the influence of *NF-κB* rs7665090 variant in gene expression patterns. It is worth mentioning that, in a previous work, our group identified an association between *TNIP1* and *TNFAIP3* with SSc, genes that are known negative regulators of the NF-κB signaling pathway, suggesting that *NF-κB* disturbances might have a role in SSc susceptibility [13].

Our single-marker analyses also revealed an association of *PLCL2* rs1372072 (OR = 1.23, 95% CI 1.12 to 1.33, *P*_adj_ = 7.22 × 10^−5^) with SSc. Phospholipase C (PLC) proteins are important mediators of the calcium-protein kinase C signaling pathway known to be expressed in hematopoietic cells, which play a role in B-cell receptor signaling and other immune responses [24]. The *PLCL2 rs4535211* variant has been reported in RA and psoriatic arthritis. The variant was associated with RA risk, whereas in psoriatic arthritis it was found to be protective [25,26]. According to HapMap data, rs1372072 and rs4535211 are in high LD (D′ = 0.90) in a block that contains the *PLCL2* gene although not in phase (R^2^ = 0.44). Therefore, our findings imply that this locus might contribute to several AIDs; this is an observation that must be confirmed by denser mapping and additional functional experiments to further increase our understanding of the role of *PLCL2* in AIDs and SSc.

We followed up two other genes (*DDX6* and *IL12A*) for which signals of association with SSc were recently described by another study [12] and because we observed for these 2 genes a nominal association in the first step of our own study for variants harbored by the same genes even if the results of the first step were not confirmed in our combined analyses. For these additional SNPs that are a common variant of *DDX6* and a rare variant of *IL12A*, we could not confirm the signal of association in our sample, ruling out independent replication. When a meta-analysis was performed, no association was observed for *DDX6* rs7130875, but the original association observed for *IL12A* rs77583790 was strengthened, thus confirming this latter locus.

As compared with linkage analyses, association studies have much less power in cases of different mutations acting in different families; however, they are far more sensitive than family studies to study complex polygenic associations. In such diseases, the phenotype is associated with the joint affects of several weakly contributing variants across various loci. However, GWASs have received criticism and skepticism because of the structure of the knowledge it has produced, which contrasts with the highly penetrant Mendelian genetic discoveries. Indeed, interpreting and understanding the molecular mechanisms related to non-coding variants are challenging given the diversity of non-coding functions, the incomplete annotation of regulatory elements, and the probable still uncovered mechanisms of regulatory control. If some studies, through extensive experimental work, uncovered some molecular mechanisms responsible for disease association [27], far more disease-associated variants remain uncharacterized. However, expanding the catalog of non-coding variants associated with human diseases is still meaningful. Indeed, several promises have emerged; for example, the application to GWAS data of gene-set enrichment analyses has provided meaningful data regarding some regulatory mechanisms [28]. The new and complementary approaches, including integration of annotation, higher resolution, and functional maps, may provide the basis for deciphering the effects of non-coding variants in pathways or cell types taking into account the weak additive interactions discovered on GWAS and the potential epistatic interactions between the variants. Such complementary strategies might finally improve the interpretation of disease-associated variants and may help identify the most likely causal regulator. These post-genomic functional studies are eagerly awaited in order to try to elucidate the biological consequences of non-coding variants.

Herein, we identified a novel epistatic interaction between *NF-κB* and *IRF8* genes, which may contribute to SSc susceptibility. This gene-gene interaction might explain the small OR obtained in our genetic association studies, which increase our understanding of missing heritability. Although the biological consequences of this interaction remain unknown, our results highlight the importance of combined analysis of genetic associations in candidate gene studies.

Taken together, our data support the shared genetic bases that are involved in SSc and PBC susceptibility. Of note, we highlight the participation of genes that are related to antigen recognition mediated by TLR and B cells, emphasizing their role in SSc susceptibility. In our mRNA expression analysis, we used resting PBMCs; hence, it is suggested that further studies use PBMC activators to enhance the expression signal.

## Conclusions

Although the exact role played by *PLCL2*, *NF-κB*, and *IRF8* is yet to be determined, we provide convincing evidence which highlights the importance of these genes in SSc. Identification of new variants, interactions between unlinked loci, and epistatic effects may help us to better understand the intricate processes of heterogeneity of AIDs.

## Additional files

Additional file 1: Table S1. List with detailed informations about the 16 tag SNPs included in the study. SNP – single nucleotide polymorphisms; BP – base pairs ; variation – major allele > minor allele. Additional file 2: Table S2. Analysis of the 13 remaining tag single-nucleotide polymorphisms (SNPs) in the combined Caucasian populations (French and Italian). ^†^After Bonferroni correction. ACA+, anti-centromere antibody; CI, confidence interval; dcSSc, diffuse cutaneous systemic sclerosis; lcSSc, limited cutaneous systemic sclerosis; MAF, minor allele frequency; n, number of pooled patients analysed; NA, not applicable; OR, odds ratio; SSc, systemic sclerosis; Topo I+, anti-topoisomerase I antibody. Additional file 3: Table S3. Results of the population meta-analysis. We used the original studies together with the French data provided in our study and looked at single-nucleotide polymorphisms (SNPs) rs1598859, rs7130875, rs11642873, and rs77583790.

## Abbreviations

AID: autoimmune disease
CI: confidence interval
GWAS: genome-wide association study
IFIT1: type I interferon
IFN-γ: interferon-gamma
lcSSc: limited cutaneous systemic sclerosis
LD: linkage disequilibrium
MAF: minor allele frequency
NF-κB: nuclear factor-kappa-B
OR: odds ratio
PBC: primary biliary cirrhosis
PBMC: peripheral blood nuclear cell
PF: pulmonary fibrosis
PLC: phospholipase C
SNP: single-nucleotide polymorphism
SSc: systemic sclerosis
TGF-β: transforming growth factor-beta
TLR: Toll-like receptor
