# Supplementary material for: Identification of NF-κB and PLCL2 as new susceptibility genes and highlights on a potential role of IRF8 through interferon signature modulation in systemic sclerosis
Source: Arthritis Res Ther. 2015 Mar 21;17(1):71. doi: 10.1186/s13075-015-0572-y (PMC4422604; doi:10.1186/s13075-015-0572-y)
Supplement: Additional file 1: Table S1. — List with detailed informations about the 16 tag SNPs included in the study. SNP – single nucleotide polymorphisms; BP – base pairs ; variation – major allele > minor allele. [file 13075_2015_572_MOESM1_ESM.doc]

**Sup-Table 1. List with detailed information about the 16 tag SNPs included in the study**

| **Chromosome** | **SNP** | **BP** | **Gene** | **Variation** | | **Putative Function** |
| --- | --- | --- | --- | --- | --- | --- |
| 1 | rs10752747 | 2514775 | *MMEL1* | G>T | phosphate metabolism and homeostasis | |
| 1 | rs12134279 | 196047821 | *DENND1B* | C>T | associated with asthma | |
| 3 | rs1372072 | 16930263 | *PLCL2* | C>T | expressed in skeletal muscle | |
| 3 | rs2293370 | 120702624 | *TIMMDC1* | C>T | regulatory signals for T lymphocytes | |
| 3 | rs485499 | 161228557 | *IL12A* | T>C | involved in Th1/Th2 cell differentiation | |
| 4 | rs7665090 | 103770651 | *NF-kB* | A>G | pleiotropic transcription factor | |
| 5 | rs860413 | 35978799 | *IL7R* | T>G | t-cell activation (SCID) | |
| 7 | rs6974491 | 37341035 | *ELMO1* | G>A | promotes phagocytosis | |
| 11 | rs6421571 | 118248982 | *DDX6* | C>T | RNA helicase in P-bodies | |
| 12 | rs1800693 | 6310270 | *TNFRSF1A* | A>G | activates NF-κB mediates apoptosis | |
| 14 | rs911263 | 67823346 | *RAD51B* | A>G | involved in DNA repair and recombination | |
| 14 | rs8017161 | 102632948 | *TNFAIP2* | G>A | induced by TNFα | |
| 16 | rs11117432 | 84576772 | *IRF8* | G>A | transcription factor of IFN | |
| 17 | rs7208487 | 34796975 | *FBXL20* | T>G | interact with ubiquitination targets | |
| 19 | rs3745516 | 55618554 | *SPIB* | G>A | lymphoid-specific enhancer | |
| 22 | rs968451 | 38000797 | *SNORD43* | G>T | noncoding small nucleolar RNA | |

* SNP – single nucleotide polymorphisms; BP – base; variation – major allele frequency > minor allele frequency.
