# Supplementary material for: Identification of NF-κB and PLCL2 as new susceptibility genes and highlights on a potential role of IRF8 through interferon signature modulation in systemic sclerosis
Source: Arthritis Res Ther. 2015 Mar 21;17(1):71. doi: 10.1186/s13075-015-0572-y (PMC4422604; doi:10.1186/s13075-015-0572-y)
Supplement: Additional file 2: Table S2. — Analysis of the 13 remaining tag single-nucleotide polymorphisms (SNPs) in the combined Caucasian populations (French and Italian). †After Bonferroni correction. ACA+, anti-centromere antibody; CI, confidence interval; dcSSc, diffuse cutaneous systemic sclerosis; lcSSc, limited cutaneous systemic sclerosis; MAF, minor allele frequency; n, number of pooled patients analysed; NA, not applicable; OR, odds ratio; SSc, systemic sclerosis; Topo I+, anti-topoisomerase I antibody. [file 13075_2015_572_MOESM2_ESM.doc]

**Sup-Table 2.** Analysis of the 13 remaining tag SNPs in the combined Caucasian populations (French, Italian)

| **SNP, phenotype (n)** | **MAF §** | **Genotype Distribution** | | | **P** | **Padj†** | **OR (95% CI)** |
| --- | --- | --- | --- | --- | --- | --- | --- |
| **MMEL1 rs10752747** | **T** | **TT (%)** | **TG (%)** | **GG (%)** |  |  |  |
| SSc (1586) | 0.31 | 9.20 | 43.63 | 47.16 | 0.62 | NS | 0.97 (0.89-1.07) |
| dcSSc (441) | 0.31 | 7.93 | 42.85 | 49.20 | 0.17 | NS | 0.89 (0.77-1.04) |
| SSc. Topo I+ (421) | 0.31 | 7.83 | 37.29 | 54.86 | 0.004 | 0.02 | 0.79 (0.67-0.92) |
| lcSSc (1018) | 0.31 | 10.31 | 43.61 | 46.07 | 0.47 | NS | 1.03 (0.93-1.15) |
| SSc. ACA+ (607) | 0.31 | 10.21 | 43.82 | 45.96 | 0.58 | NS | 1.03 (0.91-1.18) |
| Pulmonary Fibrosis (561) | 0.31 | 9.80 | 43.31 | 46.88 | 0.97 | NS | 0.99 (0.87-1.14) |
| Controls (3577) | 0.31 | 9.70 | 43.80 | 46.49 | NA | NS | NA |
| **DENND1B rs12134279** | **T** | **TT (%)** | **TC (%)** | **CC (%)** |  |  |  |
| SSc (1600) | 0.22 | 4.5 | 36.12 | 59.37 | 0.67 | NS | 1.02 (0.92-1.12) |
| dcSSc (441) | 0.21 | 7.93 | 42.85 | 49.20 | 0.16 | NS | 0.88 (0.74-1.05) |
| SSc. Topo I+ (421) | 0.22 | 5.22 | 32.54 | 62.23 | 0.58 | NS | 0.95 (0.80-1.13) |
| lcSSc (1031) | 0.22 | 4.75 | 38.60 | 56.64 | 0.09 | 0.68 | 1.10 (0.98-1.23) |
| SSc. ACA+ (611) | 0.22 | 4.25 | 39.27 | 56.46 | 0.21 | NS | 1.09 (0.94-1.26) |
| Pulmonary Fibrosis (565) | 0.22 | 4.77 | 35.75 | 59.46 | 0.70 | NS | 1.02 (0.88-1.19) |
| Controls (3592) | 0.22 | 4.70 | 34.82 | 60.46 | NA | NA | NA |
| **TIMMDC1 rs2293370** | **T** | **TT (%)** | **TC (%)** | **CC (%)** |  |  |  |
| SSc (1598) | 0.18 | 3.19 | 28.84 | 67.95 | 0.04 | 0.70 | 0.89 (0.80-0.99) |
| dcSSc (443) | 0.19 | 4.28 | 25.50 | 70.20 | 0.11 | 0.78 | 0.86 (0.71-1.03) |
| SSc. Topo I+ (420) | 0.19 | 4.52 | 28.09 | 67.38 | 0.62 | NS | 0.95 (0.79-1.14) |
| lcSSc (1025) | 0.18 | 2.63 | 30.43 | 66.92 | 0.12 | 0.90 | 0.90 (0.79-1.02) |
| SSc. ACA+ (610) | 0.19 | 3.27 | 29.50 | 67.21 | 0.27 | NS | 0.91 (0.78-1.07) |
| Pulmonary Fibrosis (564) | 0.19 | 3.72 | 28.64 | 67.73 | 0.31 | NS | 0.91 (0.78-1.08) |
| Controls (3606) | 0.19 | 3.63 | 31.25 | 65.11 | NA | NA | NA |
| **IL12A rs485499** | **C** | **CC (%)** | **CT (%)** | **TT (%)** |  |  |  |
| SSc (1605) | 0.35 | 10.59 | 45.17 | 44.23 | 0.008 | 0.13 | 0.89 (0.81-0.97) |
| dcSSc (446) | 0.36 | 11.43 | 47.30 | 41.25 | 0.57 | NS | 0.96 (0.83-1.11) |
| SSc. Topo I+ (425) | 0.36 | 10.85 | 47.76 | 41.64 | 0.59 | NS | 0.96 (0.83-1.12) |
| lcSSc (1031) | 0.35 | 9.69 | 44.13 | 46.16 | 0.001 | 0.01 | 0.84 (0.76-0.94) |
| SSc. ACA+ (614) | 0.35 | 9.44 | 42.83 | 47.71 | 0.001 | 0.01 | 0.81 (0.71-0.92) |
| Pulmonary Fibrosis (570) | 0.36 | 11.05 | 47.01 | 41.92 | 0.38 | NS | 0.94 (0.83-1.08) |
| Controls (3577) | 0.36 | 12.04 | 48.08 | 40.08 | NA | NA | NA |
| **IL7R rs860413** | **G** | **GG (%)** | **GT (%)** | **TT (%)** |  |  |  |
| SSc (1611) | 0.27 | 7.44 | 37.80 | 54.74 | 0.09 | NS | 0.92 (0.84-1.01) |
| dcSSc (441) | 0.28 | 7.70 | 38.77 | 54.87 | 0.43 | NS | 0.94 (0.80-1.10) |
| SSc. Topo I+ (425) | 0.28 | 6.58 | 36.70 | 56.70 | 0.07 | 0.50 | 0.86 (0.73-1.01) |
| lcSSc (1033) | 0.28 | 7.93 | 37.07 | 54.98 | 0.20 | NS | 0.93 (0.83-1.04) |
| SSc. ACA+ (614) | 0.28 | 8.46 | 38.11 | 53.42 | 0.81 | NS | 0.98 (0.86-1.13) |
| Pulmonary Fibrosis (568) | 0.28 | 7.21 | 38.55 | 54.22 | 0.30 | NS | 0.93 (0.81-1.07) |
| Controls (3609) | 0.27 | 7.67 | 40.59 | 51.73 | NA | NA | NA |
| **ELMO1 rs6974491** | **A** | **AA (%)** | **AG (%)** | **GG (%)** |  |  |  |
| SSc (1594) | 0.15 | 2.32 | 28.04 | 69.63 | 0.03 | 0.62 | 1.13 (1.01-1.26) |
| dcSSc (442) | 0.15 | 1.58 | 27.37 | 71.04 | 0.75 | NS | 1.03 (0.85-1.25) |
| SSc. Topo I+ (422) | 0.15 | 1.89 | 26.06 | 72.03 | 0.74 | NS | 1.03 (0.85-1.26) |
| lcSSc (1024) | 0.15 | 2.53 | 28.61 | 68.84 | 0.01 | 0.07 | 1.19 (1.04-1.36) |
| SSc. ACA+ (611) | 0.15 | 2.29 | 26.02 | 71.68 | 0.46 | NS | 1.07 (0.90-1.26) |
| Pulmonary Fibrosis (568) | 0.15 | 2.28 | 27.11 | 70.59 | 0.34 | NS | 1.09 (0.91-1.29) |
| Controls (3592) | 0.14 | 2.25 | 25.22 | 72.52 | NA | NA | NA |
| **DDX6 rs6421571** | **T** | **TT (%)** | **TC (%)** | **CC (%)** |  |  |  |
| SSc (1599) | 0.17 | 3.18 | 26.76 | 70.04 | 0.15 | NS | 0.92 (0.83-1.03) |
| dcSSc (440) | 0.18 | 2.72 | 27.04 | 70.22 | 0.27 | NS | 0.90 (0.75-1.09) |
| SSc. Topo I+ (420) | 0.18 | 2.85 | 26.90 | 70.23 | 0.32 | NS | 0.91 (0.75-1.10) |
| lcSSc (1029) | 0.17 | 3.20 | 26.53 | 70.26 | 0.19 | NS | 0.92 (0.80-1.05) |
| SSc. ACA+ (614) | 0.17 | 2.60 | 27.03 | 70.35 | 0.16 | NS | 0.89 (0.76-1.05) |
| Pulmonary Fibrosis (561) | 0.18 | 2.67 | 27.80 | 69.51 | 0.35 | NS | 0.92 (0.78-1.09) |
| Controls (3601) | 0.17 | 2.66 | 30.13 | 67.20 | NA | NA | NA |
| **TNFRSF1A rs1800693** | **G** | **GG (%)** | **GA (%)** | **AA (%)** |  |  |  |
| SSc (1589) | 0.40 | 14.97 | 48.14 | 36.87 | 0.19 | NS | 0.94 (0.87-1.03) |
| dcSSc (440) | 0.40 | 14.31 | 49.09 | 36.59 | 0.40 | NS | 0.94 (0.81-1.09) |
| SSc. Topo I+ (418) | 0.40 | 14.59 | 50.95 | 34.44 | 0.76 | NS | 0.98 (0.84-1.13) |
| lcSSc (1018) | 0.40 | 15.61 | 47.83 | 36.54 | 0.42 | NS | 0.96 (0.87-1.06) |
| SSc. ACA+ (605) | 0.40 | 14.38 | 48.26 | 37.35 | 0.19 | NS | 0.92 (0.81-1.04) |
| Pulmonary Fibrosis (559) | 0.41 | 16.99 | 49.37 | 33.63 | 0.41 | NS | 1.06 (0.93-1.20) |
| Controls (3598) | 0.40 | 16.62 | 47.44 | 35.93 | NA | NA | NA |
| **RAD51B rs911263** | **G** | **GG (%)** | **GA (%)** | **AA (%)** |  |  |  |
| SSc (1590) | 0.34 | 10.88 | 43.71 | 45.40 | 0.20 | NS | 0.94 (0.86-1.03) |
| dcSSc (439) | 0.34 | 13.43 | 39.17 | 47.38 | 0.56 | NS | 0.96 (0.82-1.11) |
| SSc. Topo I+ (416) | 0.34 | 11.77 | 36.29 | 51.92 | 0.01 | 0.09 | 0.82 (0.70-0.96) |
| lcSSc (1020) | 0.34 | 9.70 | 45.19 | 45.09 | 0.13 | 0.90 | 0.92 (0.83-1.02) |
| SSc. ACA+ (608) | 0.34 | 8.88 | 46.05 | 45.06 | 0.12 | 0.83 | 0.90 (0.79-1.03) |
| Pulmonary Fibrosis (559) | 0.34 | 16.99 | 49.37 | 33.63 | 0.07 | 0.50 | 0.88 (0.77-1.01) |
| Controls (3592) | 0.33 | 11.19 | 45.60 | 43.20 | NA | NA | NA |
| **TNFAIP2 rs8017161** | **A** | **AA (%)** | **AG (%)** | **GG (%)** |  |  |  |
| SSc (1596) | 0.40 | 16.41 | 48.93 | 34.64 | 0.48 | NS | 1.03 (0.94-1.14) |
| dcSSc (437) | 0.40 | 17.62 | 49.42 | 32.95 | 0.23 | NS | 1.10 (0.95-1.27) |
| SSc. Topo I+ (422) | 0.40 | 17.06 | 47.63 | 35.30 | 0.61 | NS | 1.04 (0.90-1.21) |
| lcSSc (1029) | 0.40 | 14.48 | 48.97 | 35.56 | 0.99 | NS | 1.00 (0.90-1.11) |
| SSc. ACA+ (612) | 0.40 | 16.66 | 47.38 | 35.94 | 0.78 | NS | 1.02 (0.90-1.16) |
| Pulmonary Fibrosis (564) | 0.40 | 16.31 | 50.53 | 33.15 | 0.33 | NS | 1.07 (0.94-1.22) |
| Controls (2299) | 0.39 | 16.35 | 46.88 | 36.75 | NA | NA | NA |
| **FBXL20 rs7208487** | **G** | **GG (%)** | **GT (%)** | **TT (%)** |  |  |  |
| SSc (1598) | 0.18 | 2.69 | 27.40 | 69.89 | 0.03 | 0.54 | 0.89 (0.79-0.99) |
| dcSSc (440) | 0.18 | 3.18 | 28.40 | 68.40 | 0.58 | NS | 0.95 (0.79-1.14) |
| SSc. Topo I+ (419) | 0.18 | 3.57 | 27.44 | 68.97 | 0.59 | NS | 0.95 (0.79-1.15) |
| lcSSc (1027) | 0.18 | 2.53 | 27.06 | 70.39 | 0.03 | 0.24 | 0.87 (0.76-0.99) |
| SSc. ACA+ (611) | 0.18 | 1.80 | 25.85 | 72.66 | 0.004 | 0.03 | 0.78 (0.66-0.92) |
| Pulmonary Fibrosis (561) | 0.18 | 3.03 | 25.84 | 71.12 | 0.08 | 0.55 | 0.86 (0.72-1.02) |
| Controls (3597) | 0.18 | 3.69 | 28.88 | 67.41 | NA | NA | NA |
| **SPIB rs3745516** | **A** | **AA (%)** | **GA (%)** | **GG (%)** |  |  |  |
| SSc (1606) | 0.26 | 6.84 | 37.48 | 55.66 | 0.21 | NS | 0.94 (0.86-1.04) |
| dcSSc (440) | 0.26 | 3.18 | 28.40 | 68.40 | 0.51 | NS | 0.95 (0.81-1.11) |
| SSc. Topo I+ (424) | 0.26 | 6.13 | 38.20 | 55.66 | 0.25 | NS | 0.91 (0.77-1.07) |
| lcSSc (1027) | 0.26 | 2.53 | 27.06 | 70.39 | 0.57 | NS | 0.97 (0.87-1.08) |
| SSc. ACA+ (614) | 0.26 | 7.32 | 38.59 | 54.07 | 0.79 | NS | 0.98 (0.86-1.13) |
| Pulmonary Fibrosis (565) | 0.26 | 5.84 | 39.46 | 54.69 | 0.42 | NS | 0.94 (0.82-1.09) |
| Controls (3592) | 0.26 | 7.62 | 37.97 | 54.39 | NA | NA | NA |
| **SNORD43 rs968451** | **T** | **TT (%)** | **TG (%)** | **GG (%)** |  |  |  |
| SSc (1606) | 0.15 | 2.05 | 27.39 | 70.54 | 0.35 | NS | 1.06 (0.94-1.19) |
| dcSSc (444) | 0.15 | 2.02 | 25.90 | 72.07 | 0.87 | NS | 0.98 (0.81-1.20) |
| SSc. Topo I+ (426) | 0.15 | 1.87 | 24.17 | 73.94 | 0.55 | NS | 0.94 (0.77-1.15) |
| lcSSc (1032) | 0.15 | 1.84 | 27.51 | 70.63 | 0.35 | NS | 1.07 (0.93-1.22) |
| SSc. ACA+ (616) | 0.15 | 1.29 | 28.08 | 70.61 | 0.60 | NS | 1.05 (0.88-1.24) |
| Pulmonary Fibrosis (567) | 0.15 | 1.94 | 25.74 | 72.31 | 0.82 | NS | 0.98 (0.82-1.17) |
| Controls (3603) | 0.15 | 2.24 | 25.83 | 71.91 | NA | NA | NA |

* SNPs – single nucleotide polymorphisms; (n) refers to the number of pooled populations analysis; SSc = systemic sclerosis; dcSSc = diffuse cutaneous SSc; Topo I+ = anti-topoisomerase I antibody; lcSSc = limited cutaneous SSc; ACA+ = anticentromere antibody; NA = not applicable; OR = odds ratio.

MAF § Minor allele frequency (according to Cochran-Mantel-Haenszel test for the combined European Caucasian populations).

**†** After Bonferroni correction; 95% CI - 95% confidence interval.
